# An Efficient Superframe Structure with Optimal Bandwidth Utilization and Reduced Delay for Internet of Things Based Wireless Sensor Networks

**DOI:** 10.3390/s20071971

**Published:** 2020-04-01

**Authors:** Sangrez Khan, Ahmad Naseem Alvi, Muhammad Awais Javed, Byeong-hee Roh, Jehad Ali

**Affiliations:** 1 Department of Electrical and Computer Engineering, COMSATS University Islamabad (CUI), Islamabad 45550, Pakistan; khansangrez@gmail.com (S.K.); naseem_alvi@comsats.edu.pk (A.N.A.); 2 Department of Computer Engineering, Ajou University, Suwon 16499, Korea; bhroh@ajou.ac.kr (B.-h.R.); jehadali@ajou.ac.kr (J.A.)

**Keywords:** Internet of Things, wireless sensor networks, superframe structure

## Abstract

Internet of Things (IoT) is a promising technology that uses wireless sensor networks to enable data collection, monitoring, and transmission from the physical devices to the Internet. Due to its potential large scale usage, efficient routing and Medium Access Control (MAC) techniques are vital to meet various application requirements. Most of the IoT applications need low data rate and low powered wireless transmissions and IEEE 802.15.4 standard is mostly used in this regard which offers superframe structure at the MAC layer. However, for IoT applications where nodes have adaptive data traffic, the standard has some limitations such as bandwidth wastage and latency. In this paper, a new superframe structure is proposed that is backward compatible with the existing parameters of the standard. The proposed superframe overcomes limitations of the standard by fine-tuning its superframe structure and squeezing the size of its contention-free slots. Thus, the proposed superframe adjusts its duty cycle according to the traffic requirements and accommodates more nodes in a superframe structure. The analytical results show that our proposed superframe structure has almost 50% less delay, accommodate more nodes and has better link utilization in a superframe as compared to the IEEE 802.15.4 standard.

## 1. Introduction

Internet of Things (IoT) is an emerging technology that has been highly attracted due to its wide utility in diverse applications since last decade. IoT is used to connect physical objects with the Internet and has plenty of applications for sensor data monitoring and information analytic [1,2,3]. In critical scenarios such as natural disasters like floods, earthquakes, and tsunamis, etc., the consequences are catastrophic and need some reliable, timely and coordinated response to overcome the damage. In this regard, the IEEE 802.15.4 based IoT plays a vital role to provide the connectivity among IoT nodes [4,5,6,7].

The above mentioned critical applications demand better throughput, reduced power consumption, and guaranteed data delivery with permitted latency [8]. Physical and Medium Access Control (MAC) layers protocols in addition to routing protocols are designed to meet critical Quality of Service (QoS) requirements of IoT applications [9,10].

IoT is a key part of future smart cities and Wireless Sensor Networks (WNSs) are its core components, which are deployed in different applications to create a Wireless Personal Area Network (WPAN). To facilitate the transmissions from various IoT nodes, it is vital to design an efficient MAC protocol. Various MAC protocols traditionally used for WSNs such as LoRaWAN [11,12], and Symphony Link [13] can be used for IoT applications but they suffer from long delay in the presence of adaptive data traffic. Energy is one of the major constraint in WSNs.

Institute of Electrical and Electronics Engineering (IEEE) has developed 802.15.4 standard for low data rate and low power Wireless Personal Area Networks (WPAN). IEEE 802.15.4 standard offers a very low duty cycle even less than 0.1% and is highly attracted by such devices having low power constrainsts such as WSNs [14].

**Figure 1:**
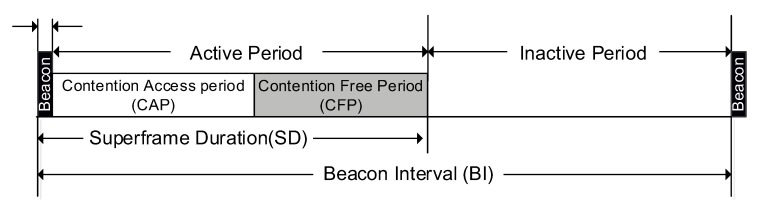
IEEE 802.15.4 Beacon enabled mode Superframe format.

IEEE 802.15.4 operates at three different frequency bands such as 868 MHz, 915 MHz, and 2.4 GHz. The standard works either in a *Beacon* enabled or *Non-Beacon* enabled mode. The Beacon enabled mode is divided into two main sections, *active* and *inactive* period, as shown in Figure 1. All WSN nodes communicate during an active period and remain in sleep mode during the later inactive period to conserve energy. The active period of Beacon enabled mode consists of Contention Access Period (CAP) and optional Contention Free Period (CFP). Each Superframe in this mode is divided into 16 equal duration time slots. One or more slots are reserved for the Beacon frame because its size may vary due to several remaining data frames for the associated nodes. The Beacon frame is generated by the PAN coordinator and contains information about frame structure, next Beacon, network, and sending messages.

The CAP consists of maximum of 16 or minimum of 9 slots. In CAP, nodes contend to access medium by following the slotted CSMA/CA mechanism. On the other hand, the maximum number of slots in CFP can be up to 7 and are known as Guaranteed Time Slots (GTS). Nodes having critical data requests are allocated Guaranteed Time Slot (GTS) by the coordinator. The nodes that are allocated GTS can explicitly carry out communication during their allocated period to the PAN coordinator. However, this standard has some limitations for GTS allocations.

1. The cumulative delay from GTS allocation till transferring of data causes a significant delay, which is appropriate for time-sensitive WSN applications.
2. Due to a limited number of CAP time slots maximum of 7 nodes can be allocated GTS.

These constraints along with the performance of the standard are evaluated and analyzed by many researchers in different prospects of application scenarios. CAP performance of the superframe structure of the standard is evaluated in different prospects on all frequency bands [15,16].

CFP performance of the standard is also analyzed and some new scenarios are proposed to improve the performance of the standard in different prospects. Multi-Factor Dynamic GTS Allocation Scheme (MFDGAS) in [17] proposed CFP slots allocation to nodes by considering their data traffic, communication delay and slot size requirement. This improves the GTS utilization of CFP at the cost of fairness. However, it does not address latency issues and QoS is compromised.

In [18], Advanced GTS Scheduling (AGS) is proposed for industrial applications. Authors claim that AGS improves link utilization and also chances of collisions during GTS requests are avoided. B. Lee et al. [19] proposed a priority-based algorithm for adaptive superframe adjustment and GTS allocation (PASAGA) for IoT applications. The algorithm prioritizes GTS to sensitive data as compared to other data traffic. The authors claim that PASAGA improves bandwidth utilization and improves delay for sensitive data in comparison to the standard.

In [20], authors proposed an optimal relay selection technique for IEEE 802.15.4 based sensor to sink communications. The authors also propose an efficient channel access mechanism to improve the network throughput and reduce packet collisions resulting in lower energy consumption of the sensor nodes. In [21], a novel medium access protocol for the IEEE 802.15.4 Time-slotted Channel Hopping (TSCH) based wireless sensor networks is proposed. The proposed protocol uses Enhanced Beacons (EBs) based scheduling approach to minimize the collisions due to simultaneous transmissions of the sensor nodes. As compared to the centralized schemes, the proposed autonomous scheduling protocol improves the energy consumption and throughput.

**Figure 2:**
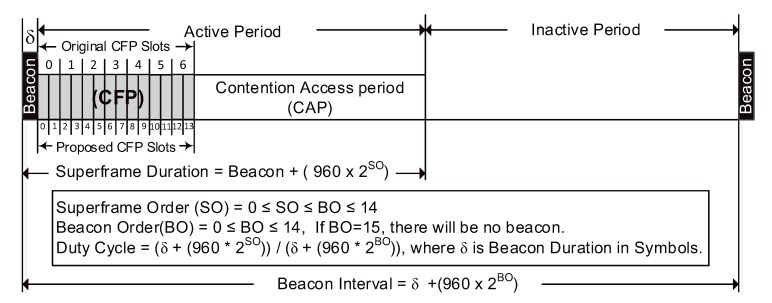
Efficient Superframe Structure.

All research related to maximizing the throughput and reduce communication delay of the traffic in CFP consider the above-mentioned scenario mentioned in the 802.15.4. standard [22]. However, these constraints were properly addressed in [23] by proposing an Efficient Superframe Structure (ESS) where CFP precedes the CAP as shown in Figure 2. This superframe structure minimized the delay in a significant manner. The link utilization has also been improved by reducing the GTS duration to half of the normal slot length.

A large number of applications offer adaptive data traffic with an adaptive duty cycle to meet QoS. One of the requirements of such applications is to avoid unnecessary delay, optimally scrutinize data requesting nodes in a superframe structure with improved link utilization. To meet these requirements, an adaptive duty cycle according to the data requests is required. Though ESS manages the delay and link utilization to some extent, however, ESS as well as standard does not meet the adaptive data requirements of GTS requesting nodes.

In this work, an Efficient Superframe Structure with Adaptive Duty Cycle $\left(ESS_{ADC}\right)$ is proposed to meet the adaptive data requirements. $ESS_{ADC}$ follows the superframe structure of ESS by preceding CFP than CAP for reduced network delay and introduces an algorithm, that allows PAN coordinator to adjust its duty cycle according to the traffic requirements.

Major contributions of $ESS_{ADC}$ are:

- $ESS_{ADC}$ adjusts active period of the superframe to improve the GTS utilization and offers better data transmission.
- Similar to ESS, GTSs have been doubled by reducing their slot size to half of the IEEE 802.15.4 standard. This helps in accommodating up to 14 GTS requesting nodes instead of 7.
- PAN coordinator scrutinizes the GTS requesting nodes by applying Shortest Job First (SJF) algorithm instead of first come first serve. This helps in reducing the network delay.
- $ESS_{ADC}$ is backward compatible with the standard and adequate with existing parameter.

The rest of the paper is organized in the following manner: Section 2 briefly describes the IEEE 802.15.4 standard by emphasizing on the GTS allocation procedure. Section 3 discusses the proposed superframe format along with the necessary modifications in the beacon frame fields. The numerical estimators for the delay and link utilization for the proposed superframe format are also presented in this section. The numerical results of our proposed scheme are compared with the ESS and the beacon-enabled IEEE 802.15.4 standard in Section 4. Finally, Section 5 concludes our work.

## 2. Overview of IEEE 802.15.4 Standard

The standard is designed for low-rate wireless personal area networks (LR-WPAN), that supports both star and peer-to-peer topology. The standard operates in both beacon and non-beacon enabled modes.

In non-beacon-enabled mode, there is no duty cycle, as there are no active and inactive periods and allows nodes to communicate in an ad-hoc manner by using an un-slotted CSMA/CA algorithm. However, in beacon-enabled mode, the standard offers superframe structure, that comprises of an active and optional inactive period with an adaptive duty cycle, that ranges from less than 0.1% up to 100%. For 100% duty cycle, there is no inactive period. An active period is known as Superframe Duration (*SD*) and it comprises of the beacon, CAP and CFP. PAN coordinator broadcasts a beacon frame and it is mandatory for all member nodes to listen this beacon message not only for time synchronization but also to get the information about the CAP, CFP, inactive period and interval between two consecutive beacons (*BI*). *SD* comprises of 16 slots, minimum 9 slots are shared between Beacon frame and CAP, whereas maximum limit of CFP is 7 in a *SD*. A complete superframe structure of IEEE 802.15.4 standard is shown in Figure 1.

The parameter values of Superframe Order (*SO*) and Beacon Order (*BO*) determines *SD* and BI as mentioned in Equations (1) and (2).

(1) $$SD=aBasesuperframeduration\times 2^{SO}$$

(2) $$BI=aBasesuperframeduration\times 2^{BO}$$

where, 0≤SO≤BO≤14.

Duty cycle (*DC*) in a beacon-enabled mode is controlled by varying the values of *SO* and *BO*. *DC* can be calculated as:

(3) $$DC=2^{BO-SO}$$

During CAP, nodes transmit their requests to their PAN coordinator and PAN coordinator assigns Guaranteed Time Slots (GTS) to nodes for data transmission during CFP.

### 2.1. GTS Allocation Procedure

The standard allocates CFP slots to only those nodes which are a member of the PAN and holds a short address. A node can determine the number of GTS required $\left(GTS^{req}\right)$ to send its data (*D*), once it knows the slot capacity by knowing the value of SO, with the help of Equation (4).

(4) $$GTS^{req}=\left|D/SC\right|$$

Here, SC is the slot capacity to carry maximum number of bits and is computed as:

(5) $$SC=960\times 2^{SO-2}$$

**Figure 3:**
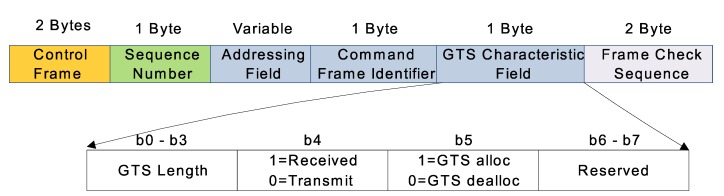
GTS request frame format of IEEE 802.15.4.

Each GTS requesting node calculates $GTS^{req}$ and send it to the coordinator in CAP. A GTS requesting frame is shown in Figure 3.

PAN Coordinator receives GTS requests only in CAP. At the end of the CAP, it evaluates the GTS requests. If GTS requests are within the available slot limits, then it allocates GTS to all the GTS requesting nodes. In case, requesting GTS are more than 7, then the PAN coordinator scrutinizes GTS on a first come first serve basis. All the scrutinized nodes are informed about their allocated CFP slots with their starting and ending slot during the next beacon frame. An increase in successful nodes increases the length of the beacon frame that reduces the CAP length. PAN coordinator also ensures to maintain the minimum CAP duration, as it should not be less than *aMinCAPLength* value. The scrutinized nodes retrieve information about their assigned CFP slots from the GTS descriptor field of the beacon frame.

### 2.2. Brief Overview of ESS

This section discusses the efficient superframe structure introduced in [23] as shown in Figure 2. In ESS, CFP precedes soon after the beacon frame and then followed by CAP. In case, there is no GTS request received by the PAN coordinator then, there is no CFP and CAP will commence right after the Beacon frame. CFP duration in ESS is similar in size as offered by the standard, however, each CFP slot duration is halved to increase the slot capacity to 14 instead of 7. The salient feature of ESS is to avoid the unnecessary CAP delay faced by a GTS requesting node in the next BI. This significantly reduces the network delay faced by the GTS requesting nodes. Besides, 14 GTS can accommodate more GTS requesting nodes to transmit their data in a BI, that improves the network throughput. The reduced delay and improved throughput in ESS is achieved at the cost of some modifications in the existing parameters of the standard.

The Superframe structure of ESS contains minimum 9 CAP slots and maximum 14 CFP slots excluding the beacon frame. The Superframe Duration of ESS $\left(SD_{ESS}\right)$ and Beacon Interval of ESS $\left(BI_{ESS}\right)$ are calculated as: depends upon the value of Superframe order (*SO*), aNumSuperframeSlot (NSS) and aBaseSlotDuration (BSD) as:

(6) $$SD_{ESS}=\delta +\left(960\times 2^{SO}\right)$$

(7) $$BI_{ESS}=\delta +\left(960\times 2^{BO}\right)$$

here, 0≤SO≤BO. Default value of *aUnitBackoffPeriod* is 20 Symbols. The Beacon Duration in symbols, δ, in Equation (8) is computed as:

(8) δ=(m+3∗n)×2(Symbols)

A large number of applications offer adaptive data traffic with an adaptive duty cycle to meet QoS. Both IEEE 802.15.4 standard and ESS do not entertain the adaptive data traffic due to their fixed *SO* and *BO* values in a superframe. This results in either not entertaining nodes optimally or poor GTS utilization in a superframe duration. When nodes have data requests which are more than the available limit, then PAN coordinator will not be able to accommodate maximum data requesting nodes. However, when data requests by each node is less than the available slot capacity, then GTS utilization is compromised. In this work, an active period of the superframe structure is fine tuned to optimally adjust the data requesting nodes in CFP.

## 3. Efficient Superframe Structures with Adaptive Duty Cycle $\left(ESS_{ADC}\right)$

In this work, an efficient superframe structure with adaptive duty cycle $\left(ESS_{ADC}\right)$ is proposed that optimally adapts the duty cycle of superframe structure of ESS to meet the adaptive GTS requirements of GTS requesting nodes. The proposed algorithm in $ESS_{ADC}$ offers reduced delay and increased GTS utilization as compared to ESS and the standard by effectively using some existing and unused parameters of the standard.

Initially all the GTS requesting nodes $\left(Node_{GTS}\right)$ are required to determine the number of GTS required $\left(GTS_{req}\right)$ to send their data. $GTS_{req}$ in the standard is calculated by knowing the requested data (DR) and data slot capacity $\left(DS_{cap}\right)$ of each slot of the superframe as:

(9) $$GTS_{req}=\left\lceil \frac{DR}{DS_{Cap}}\right\rceil$$

### 3.1. GTS Request Frame Structure

In $ESS_{ADC}$, each CFP slot is halved, which increases the GTS capacity from 7 to 14 without including any new parameter values in the standard. This doubles the GTS capacity in each SD at the cost of marginally increased computing, as each node need has to determine the data slot capacity of each CFP slot $DS_{Cap}^{ESS_{ADC}}$ with the help of following equation.

(10) $$CFP^{SLOT}=960\times 2^{SO-3}\left(bits\right)$$

All the $Node_{GTS}$ calculate their $GTS_{req}^{ESS_{ADC}}$ in $ESS_{ADC}$ as:

(11) $$GTS_{req}^{ESS_{ADC}}=\frac{DR}{DS_{Cap}^{ESS_{ADC}}}$$

Equation (11) generates a fractional number, that is transmitted by each requesting node to PAN coordinator. In this work, two unused bits b6 and b7 of each GTS requesting frame are used as shown in Figure 3. The value of these bits is determined by computing the value of *X* as:

(12) $$X=\left|\left\lceil log_{2}\left(\frac{DR}{DS_{Cap}}\right)\right\rceil \right|$$

Each requesting node fills these two bits as shown in Table 1.

These bits helps PAN coordinator to know the fractional value of data slots requested by a node. The information of these two bits are only meaningful when node either requests for one GTS or by requesting 14 GTS by sending value from b0 to b3 as 0001 and 1110 respectively. There are chances that the requested data of the node might be smaller in size as compared to the $DS_{Cap}$. Decrease of 1 in *SO*, halves the GTS capacity to its previous capacity. That’s why, the PAN coordinator needs to know about GTS utilized before decreasing its size. However, b0 to b3 value 1110 means, that a node has requested maximum available GTS. In this case, b6 and b7 will mention, how many more CFP slots, a node requires to send its data completely.

PAN coordinator at the end of CAP, accumulates all GTS requests and apply the $ESS_{ADC}$ algorithm as described in Section 3.2.

### 3.2. $ESS_{ADC}$ Algorithm

**Figure 4:**
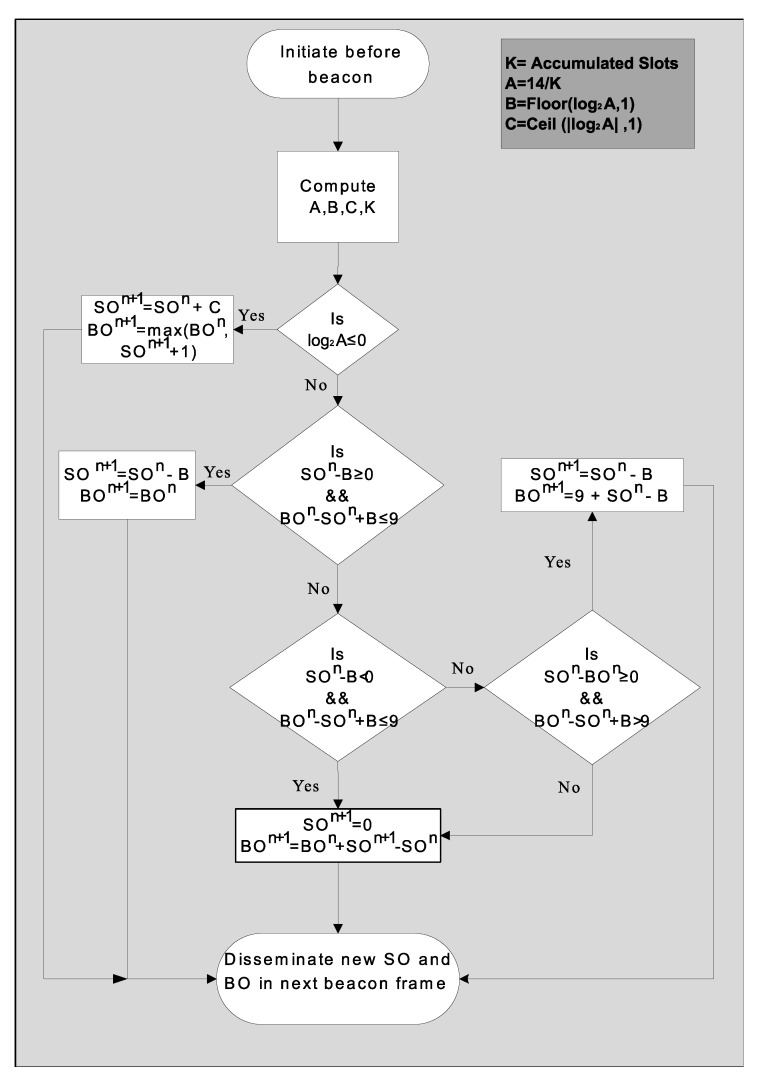
Proposed $ESS_{ADC}$ algorithm.

PAN coordinator applies $ESS_{ADC}$ algorithm by adjusting the values of SO and BO as shown in Figure 4.

After receiving all the $GTS_{req}^{ESS_{ADC}}$, PAN coordinator calculates the fractional slot requests for each node. Suppose node *S* requested *k* slots. It needs to compute the percentage of available GTS being utilized by all nodes. This will help the PAN coordinator to determine the slot usage in fractions. PAN coordinator accumulates all these slots (K) requested by all nodes *S*. The coordinator computes the ratio (A) between total available slots as compared to the GTS requested by all nodes. The proposed scheme offers 14 GTS in a superframe, so A = 14/K. PAN coordinator after computing the value of A, needs to compare it with the total number of available GTS. In case, the value of A is greater than the available GTS, then it needs to increase the duty cycle by increasing the value of SO. At the same time, the duty cycle should not increase from 50%. This increase in the duty cycle depends on the value of C calculated as:

(13) $$C=\left\lceil (\left|log_{2}A\right|,1)\right\rceil$$

If A is less than the available GTS limit, then the duty cycle may be reduced. This decrease in duty cycle should not violate the SO and BO limits as defined in the standard. At the same time, the PAN coordinator should be precise about the reduction in the duty cycle. The reduced parameters of SO and BO depends upon the value of *B*, which is determined as:

(14) $$B=\left\lfloor (log_{2}A,1)\right\rfloor$$

This algorithm helps the PAN coordinator to adapt the duty cycle of the next superframe without violating the standard limits, such as the difference between BO and SO should not increase from 9 and values of SO and BO should remain in their boundary limits.

**Figure 5:**
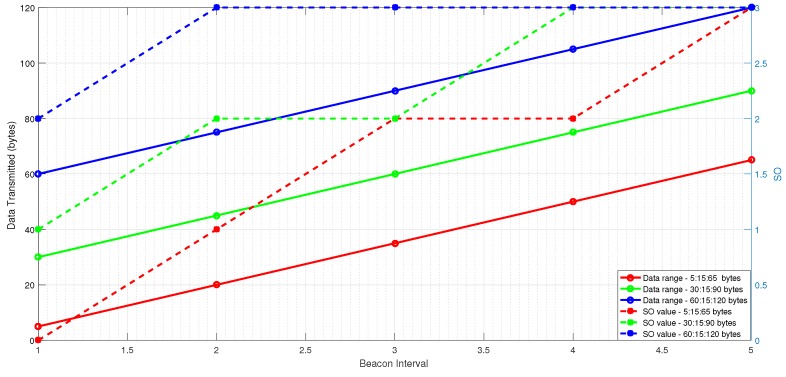
$ESS_{ADC}$ adjusting its SO against transmitted data.

Figure 5 shows that $ESS_{ADC}$ algorithm adjusts SO according to the data requests generated by the nodes for three different data ranges. The SO trend is observed for five different beacon intervals. In each beacon interval, 15 nodes send their data requests to PAN coordinator. The nodes increase their data requests with an increment of 15 bytes in the next beacon interval. It is evident from the results that, SO value becomes 0, 1, 2 and 3, when data requests of nodes are less than 16 bytes, 16–30 bytes, 31–60 bytes and 61–120 bytes respectively.

### 3.3. Nodes Selection

PAN coordinator after computing the new SO and BO, recomputes the number of CFP slots $\left(GTS^{new}\right)$ for all $node_{GTS}$ to transmit their data based on the new parameter value of SO. It scrutinizes and allocates GTS to all the $node_{GTS}$ with their $GTS^{new}$ by applying SJF, that is, by allowing nodes with less GTS requests to send their data before the other nodes. This helps more nodes to complete their transmission earlier as compared to the standard, resulting in reduced network delay at the cost of minute fairness.

In this work, similar to the standard, the duration of CAP and CFP depends solely on the value of SO. However, each slot in CFP has been halved to increase its capacity to 14 instead of 7 as mentioned in ESS. This increases the maximum capacity of GTS from 7 to 14 within a superframe structure. Data capacity of each CFP slot $\left(CFP^{SLOT}\right)$ can be determined by a node from the following equation.

(15) $$CFP^{SLOT}=960\times 2^{SO-3}\left(bits\right)$$

### 3.4. Link Utilization

It has been observed that a significant amount of bandwidth is wasted during CFP in the standard. Higher the slot size more will be the wastage. Though ESS reduces this wastage by reducing the CFP slot size to half however, it is not optimal with adaptive data traffic in each BI. If $D_{i}$ data is required to be transmitted by node *i*, then the time required to send this data $t_{d}$ to PAN coordinator is estimated as:

(16) $$t_{d}=\frac{D_{i}}{C}$$

Here *C* is the data rate through which node communicates. If $K_{i}$ is the number of CFP slots required to send $D_{i}$ data, then it is computed as:

(17) $$K_{i}=\frac{D_{i}}{N_{bps}}$$

here, $N_{bps}$ is the number of maximum bits, that can transmitted during each CFP slot and is calculated as:

(18) $$N_{bps}=15\times 2^{SO+3}$$

Larger the slot size more will be its capacity in transmitting data in a CFP. $N_{bps}$ is fixed in ESS due to its unchanged SO throughput, however in $ESS_{ADS}$, it varies due to its adjustment according to adaptive data traffic. If a node *i* requires $K_{i}$ slots in transmitting its data $D_{i}$ to PAN-coordinator, then link utilization $\left(U_{i}\right)$ for node *i* is calculated as:

(19) $$U_{i}=\frac{t_{d}}{K_{i}\times t_{s}}$$

here, $t_{s}$ is the time in seconds of each CFP slot and it is computed as

(20) $$t_{s}=15\times e^{-6}\times 2^{SO+5}$$

If *p* nodes are successfully allocated CFP slots, then the link utilization $\left(U_{CFP}\right)$, for *p* number of nodes is computed as:

(21) $$U_{CFP}=\sum_{i=1}^{p}\frac{t_{d}}{K_{i}\times t_{s}}$$

However, link utilization of same node *i* for the IEEE 802.15.4 standard ($U_{o_{i}}$) is calculated as:

(22) $$U_{o_{i}}=\frac{t_{d}}{K_{o}\times t_{o}}$$

here $k_{o}$ is number of CFP slots required to send data during CFP in current standard and $t_{o}$ is the time of each CFP slot in seconds and it is calculated as:

(23) $$t_{o}=15\times e^{-6}\times 2^{SO+4}$$

If *q* nodes have been successfully assigned CFP slots, then link utilization $U_{CFP_{o}}$ during a specific BI is calculated as:

(24) $$U_{CFP_{o}}=\sum_{i=1}^{q}\frac{t_{i}}{K_{o}\times t_{o}}$$

## 4. Numerical Analysis with Results

To analyze and evaluate the $ESS_{ADC}$ performance with IEEE 802.15.4 and ESS, the simulation environment is created by deploying 20 sensor nodes and a PAN coordinator. This simulation environment is created in MATLAB. Each node is allowed to transmit different data in each beacon interval that ranges from 20 to 300 bytes. The effects of different values of BO and SO parameters is observed for fair analysis of ESS and the standard with $ESS_{ADC}$. For better comparison of our proposed scheme with the other two, the proposed superframe structure is evaluated in different prospects, such as delay calculations, link utilization and slot(s) allocation to GTS requesting nodes. Table 2 shows a complete list of simulation parameters.

### 4.1. Delay Analysis

Transmission delay of a node is the duration, when a node has data to transmit till its successful transmission to the PAN coordinator. Suppose, a node *j* needs to send *L* amount of data just at the beginning of the beacon frame, its delay in $ESS_{ADC}$$\left(D_{ESS_{ADC}}\right)$ is calculated as:

(25) $$D_{ESS_{ADC}}^{j}=BI_{ESS}+\left(\sum_{b=1}^{b=j}K_{b}\times t_{ESS_{ADC}}\right)$$

Here,

$$t_{ESS_{ADC}}\left(sec\right)=4.8\times 10^{-4}\times 2^{SO}$$

and $K_{b-1}$ describes the total slots assigned to node *j* and previously allocated slots to the preceding nodes. ESS follows the same formula in finding its delay, however its SO is fixed whereas, $ESS_{ADC}$ adapts its SO and BO according to the data requirement. Delay of ESS and $ESS_{ADC}$ will only be same, when both have same values of SO and BO. If *Y* nodes are successfully scrutinized for GTS allocation, then accumulated delay of the PAN in $ESS_{ADC}$ ($D_{ESS_{ADC}}^{max}$) is computed as:

(26) $$D_{ESS_{ADC}}^{max}=\sum_{i=1}^{i=Y}\left[BI_{ESS_{i}}+\left(\sum_{b=1}^{b=i}K_{b}\times t_{ESS_{ADC}}\right)\right]$$

However, in IEEE 802.15.4 standard, delay of the same node *j* in transmitting its data ($D_{15.4}$) is calculated as:

(27) $$D_{15.4}=BI+SD-\left(\sum_{b=1}^{b=j}K_{b-1}\times t_{15.4}\right)$$

Here,

(28) $$t_{15.4}=9.6\times 10^{-4}\times 2^{SO}$$

If *X* nodes are successfully scrutinized for GTS allocation, then accumulated network delay of the PAN in IEEE802.15.4 standard $\left(D_{15.4}^{max}\right)$ is determined as:

(29) $$D_{15.4}^{max}=\sum_{i=1}^{i=X}\left[{\left(BI+SD\right)}_{i}-\sum_{x=1}^{x=i}K_{x-1}\times t_{15.4}\right]$$

**Figure 6:**
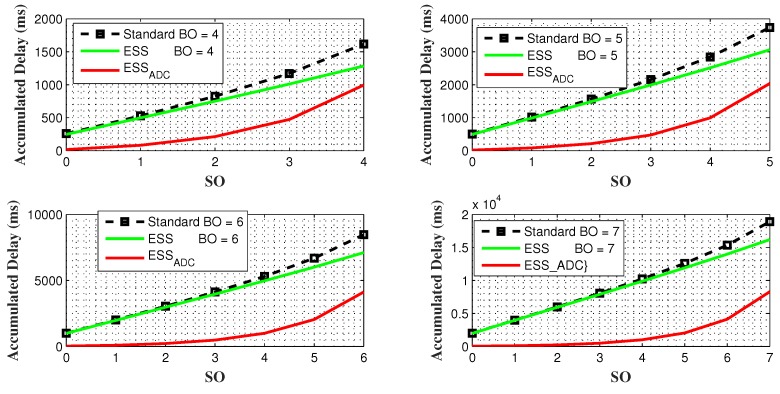
Accumulated delay comparison against all ranges of 4 different BO values.

Figure 6 comprises of 4 sub-figures that shows delay comparison of $ESS_{ADC}$ with ESS and IEEE 802.15.4 standard for four different values of BO such as 4, 5, 6, and 7. In each subplot, accumulated delay of nodes between all three schemes is evaluated for all possible values of SO against each BO, when each node has 20 bytes of data. The results verified that accumulated delay in $ESS_{ADC}$ is less than ESS and IEEE802.15.4 standard. This significant different is due to fixed SO and BO values in ESS and the standard. However, $ESS_{ADC}$ allows PAN coordinator to adapt its duty cycle to accommodate GTS requests with minimum SO and BO values. Consequently, resulting in a significant decrease in the accumulated delay as compared to other two.

**Figure 7:**
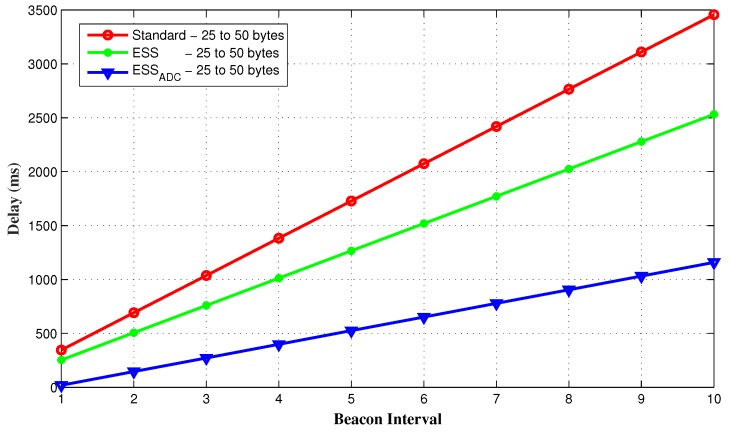
Accumulated delay for random data traffic.

The results shown in Figure 7 further verifies that accumulated delay of all nodes within the data range of 25 to 50 bytes are significantly less in the proposed $ESS_{ADC}$ as compared to the other two schemes when they both have SO = 3 and BO = 4.

### 4.2. Link Utilization and Data Transmission

**Figure 8:**
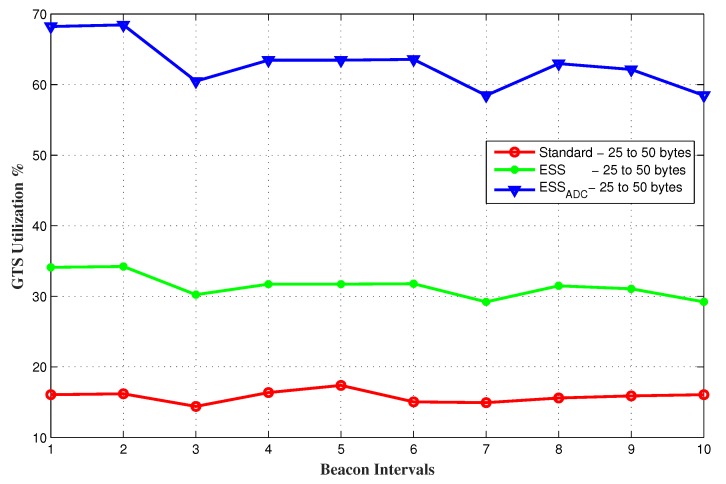
GTS utilization of the network for random data requesting nodes.

Link utilization during CFP is calculated in percentage and it is the ratio of the used bandwidth by all GTS allocated nodes to the total allocated bandwidth for all these nodes in a superframe. The results shown in Figure 8 verify that GTS utilization in $ESS_{ADC}$ is more than ESS and the standard, when nodes have random data requests in the range of 25 to 50 bytes. SO and BO values chosen for these results for both ESS and the standard are 3 and 4 respectively.

**Figure 9:**
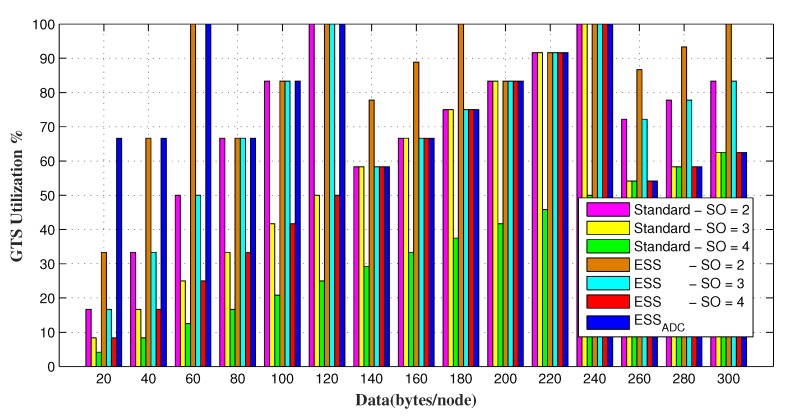
GTS utilization of the network for varying range of fixed data requesting nodes.

GTS utilization of $ESS_{ADC}$ is evaluated against three different values of *SO* of ESS and IEEE 802.15.4 standard in Figure 9. The results show that link utilization of $ESS_{ADC}$ varies on varying data requests because it adjusts its slot size according to the requesting nodes to accommodate a maximum number of nodes. As increment of 1 in *SO* value doubles the GTS duration, hence less will be the utilization. Smaller *SO* value increases the GTS utilization as a majority of its slots are filled. However, data transmission and node allocation are not optimal in smaller CFP slots.

**Figure 10:**
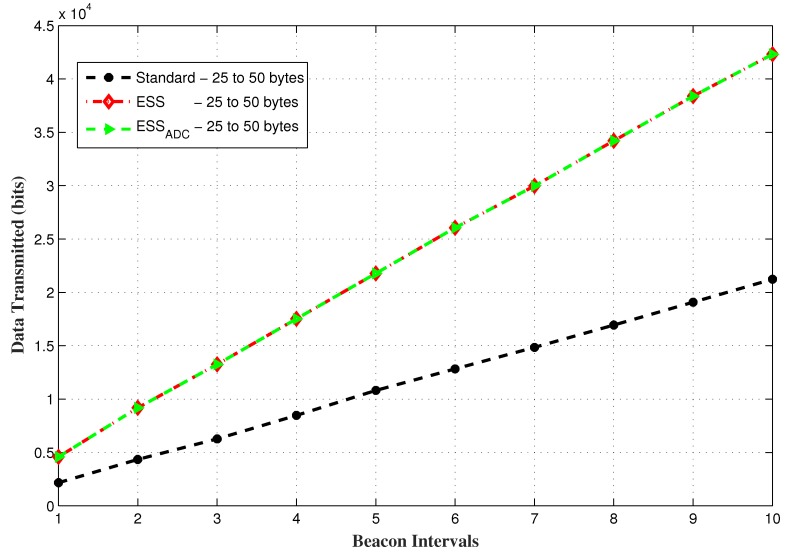
Data transmission by all nodes for random data requesting nodes.

A comparative analysis of the proposed scheme with the other two schemes in respect of data transmission, when both have SO = 3 and BO = 4 for random data range is shown in Figure 10. $ESS_{ADC}$ adjusts its slot size according to the data traffic to accommodate 14 GTS requesting nodes. Results show that the amount of data transmitted in $ESS_{ADC}$ is similar to the ESS in different beacon intervals but greater than IEEE 802.15.4 standard. This is due to the larger slot duration in ESS and IEEE 802.15.4 standard with SO = 3, which allocates a separate slot to each data requesting node, consequently capacity nodes are entertained. Maximum GTS capacity in a superframe duration in ESS and the standard is 14 and 7 respectively, that’s why ESS allows more data transmission as compared to the standard.

**Figure 11:**
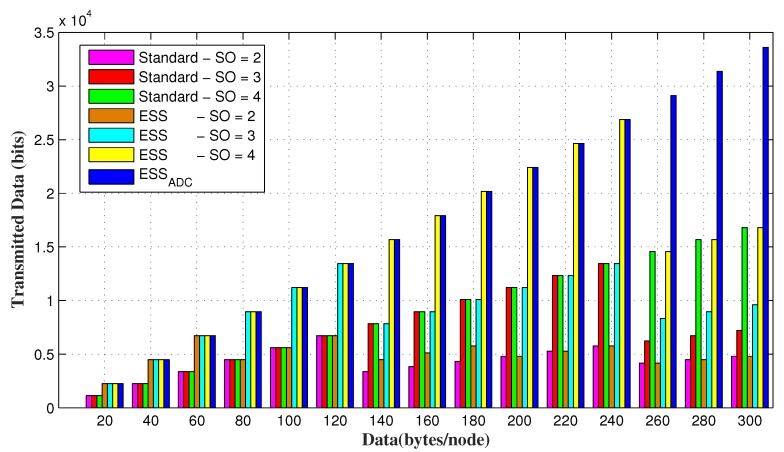
Data transmission by all nodes for varying range of fixed data requesting nodes.

Figure 11 shows, that $ESS_{ADC}$ allows nodes to send more data as compared to ESS and IEEE 802.15.4 standard for different values of *SO*, when requested data of each node increases from 240 bytes. However, when each node has less amount of data, $ESS_{ADC}$ accommodates same amount of data as allowed by ESS. This is due to the reason that duty cycle adjustment of the proposed scheme is similar to the value of ESS.

### 4.3. GTS Allocation Nodes

PAN coordinator allocates GTS to the GTS requesting nodes up to its maximum capacity. It is obvious that the increase in *SO* results in longer slot duration, that can accommodate more data request. IEEE 802.15.4 standard has only 7 GTS in a superframe duration and can adjust the maximum 7 nodes by allocating GTS. However, superframe duration of both $ESS_{ADC}$ and ESS contains 14 CFP slots by reducing each CFP slot to its half size that helps it to accommodate up to 14 GTS requesting nodes.

**Figure 12:**
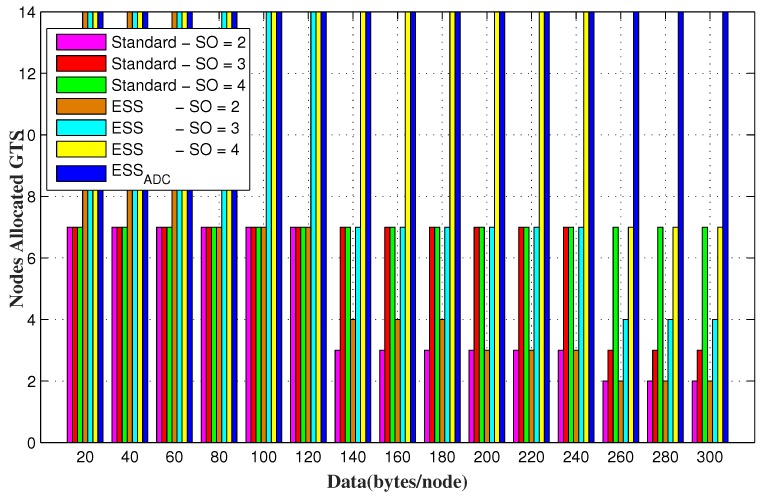
GTS allocating nodes for random data requesting nodes.

**Figure 13:**
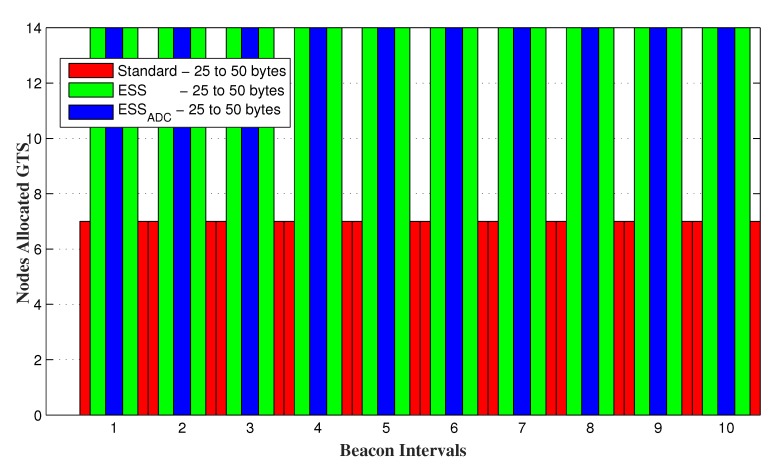
GTS allocating nodes for varying range of fixed data requesting nodes.

Figure 12 and Figure 13 shows the number of successful GTS allocating nodes for random and fixed data requests of each node respectively. It is evident from the results that $ESS_{ADC}$ and ESS allow up to 14 GTS requesting nodes to send their data in a superframe duration as compared to the IEEE 802.15.4 standard. If data requests of each GTS requesting node is less than the slot capacity then maximum nodes will be entertained, such as 7 for the standard and 14 each for $ESS_{ADC}$ and ESS as shown in Figure 13. However, Figure 12 verifies that $ESS_{ADC}$ performs better than three different SO values of ESS and the standard for adaptive data requests. The results show that ESS and the standard allows maximum nodes to send their data unless, data requests by each node is within their respective slot limit. This is due to its duty cycle adjustment, that allows $ESS_{ADC}$ to accommodate 14 nodes in a SD.

## 5. Conclusions

In this paper an efficient superframe structure $ESS_{ADC}$ of IEEE802.15.4 standard for such IoT based applications, where an adaptive duty cycle is required. The proposed scheme helps PAN coordinator to adjust its duty cycle according to data requests received from its member nodes. The proposed superframe structure is backward compatible with the standard without any inclusion in additional parameters with minute modifications in the existing parameter values. The proposed work is compared with the standard and ESS. The analytical results show that this $ESS_{ADC}$ improves delay, offer better GTS utilization with better data transmission and accommodates more number of nodes as compared to the original 802.15.4 standard and the ESS in all respects.

## Figures and Tables

**Table 1:** Parameter values describing slot utilization.

b0 − b3 (GTS Req)	Value of X	b6, b7 (Reserved Bit)	Description
0001	0	00	More than 50% slot is occupied
0001	1	01	GTS between 25% to 50% is occupied
0001	2	10	GTS between 12.5% to 25% is occupied
0001	3	11	GTS between 6.25% to 12.5% is occupied
1110	4	00	No more additional GTS required
1110	5	01	1 to 15 more GTS required
1110	6	10	16 to 31 more GTS required
1110	7	11	32 to 63 more GTS required

**Table 2:** Simulation Parameters.

Parameters	Values
Number of Nodes	21
Network Size	100 m × 100 m
Data Rate	250 Kbps
Offered Load/node (Bytes)	20:20:300
Superframe Order in ESS and 802.15.4	2:1:4
Superframe Order (initial) in $ESS_{ADC}$	0
Beacon Order in ESS and 802.15.4	3:1:5
Beacon Order (initial) in $ESS_{ADC}$	0
Duty Cycle in 802.15.4 and ESS	50%
Duty Cycle in $ESS_{ADC}$	50%
GTS Duration in 802.15.4 (sec)	$9.6\times 10^{-4}\times 2^{SO}$
GTS Duration in $ESS_{ADC}$ and ESS (sec)	$4.8\times 10^{-4}\times 2^{SO}$
